# Impact of switching from manual to automated aerobic blood culture on bacteremia diagnosis in Lao PDR

**DOI:** 10.1128/spectrum.01716-25

**Published:** 2026-03-16

**Authors:** Risara Jaksuwan, Mavuto Mukaka, Phonelavanh Phoumin, Koukeo Phommasone, Sayaphet Rattanavong, Vilada Chansamouth, Tamalee Roberts, Mayfong Mayxay, Viengmon Davong, Manivanh Vongsouvath, Elizabeth A. Ashley

**Affiliations:** 1 Lao-Oxford-Mahosot Hospital-Wellcome Trust Research Unit (LOMWRU), Microbiology Laboratory, Mahosot Hospital 251778, Vientiane, Lao PDR; 2 Mahidol-Oxford Tropical Medicine Research Unit 469893, Bangkok, Thailand; 3 Centre for Tropical Medicine and Global Health, Nuffield Department of Medicine, University of Oxford 6396, Oxford, United Kingdom; 4 Institute of Research and Education Development (IRED), University of Health Sciences, Ministry of Health 374369, Vientiane, Lao PDR; 5 Saw Swee Hock School of Public Health, National University of Singapore 37580, Singapore, Singapore; The University of Texas at Tyler, Tyler, Texas, USA

**Keywords:** blood culture, Laos, automation, microbiology, LMIC, blood volume

## Abstract

Funding for antimicrobial resistance surveillance has led to more laboratories in low-resource settings switching to automated blood culture. There is limited evidence for the impact of switching from manual to automated blood culture in low-resource tropical settings. The objective of this study was to compare the yield of clinically significant blood culture isolates between manual and automated aerobic culture methods in adults and children in a diagnostic laboratory in Lao PDR. This retrospective study analyzed blood culture data from Mahosot Hospital, Vientiane. The switch from manual (two bottles in children and adults) to automated blood culture (one pediatric or one standard aerobic bottle) took place on 8 October 2018. Blood culture results, total blood volume, time to a positive culture of a significant isolate, and method used (manual or automated) were analyzed by multiple logistic regression and survival analysis. We analyzed 34,142 blood culture episodes from 1 January 2016 to 31 December 2023, of which 12,709 (37.22%) were processed by manual methods. The median blood volumes cultured per episode in babies, children, and adults were 1.9, 4.5, and 11.2 mL for manual vs 0.9, 1.8, and 10.6 mL for automated culture. The percentage of significant pathogens detected did not differ between manual and automated methods (6.7% vs 6.3%, 95% confidence interval [CI] [0.882–1.057]). However, presumed contaminants were detected at different rates between the two methods (4.1% vs 3.9%, 95% CI [0.684–0.864]). The odds ratio (OR) of detecting a significant pathogen increased by about 2.3% per 1 mL increase in blood volume (OR = 1.023, 95% CI [1.013–1.033], *P* < 0.001), while the odds of detecting a contaminant decreased by about 13% per 1 mL increase in blood volume (OR = 0.870, 95% CI [0.854–0.886], *P* < 0.001). The automated process led to reporting of significant growth approximately 1.3 times faster than the manual process, with a hazard ratio of 1.32 and a 95% CI of 1.20–1.46. In Laos, automated processing led to reporting of significant organisms from blood cultures more quickly than manual processing, with similar percentages of significant organisms and contaminants between the two methods. Switching to one automated bottle from two manual bottles for collection had the unintended consequence of reducing the volume of blood cultured from children.

**IMPORTANCE:**

In a primary to tertiary hospital in Laos, automated processing of blood cultures did not impact on yield of significant growth but did reduce the time to growth with a significant pathogen compared to manual processing; however, switching to a single bottle led to a reduction in the total volume of blood being sent to the laboratory for culture in all age groups. Monitoring and feedback to clinicians on blood volume received may help optimize the use of blood culture. Adding another aerobic or anaerobic culture bottle for adults would be desirable but would double the cost.

## INTRODUCTION

Blood culture is the gold standard method for the detection of bacteremia (1) and is one of the most important sources of antimicrobial resistance (AMR) surveillance data globally (2). Guidelines for empiric management of sepsis are derived from blood culture results; therefore, accurate and reliable diagnosis of bloodstream infection (BSI) is essential (3). Low- and middle-income countries (LMICs) face many challenges when implementing blood culture, related to cost, logistics, and infrastructure (4). Automated blood culture systems for incubation and growth monitoring have been used in high-income countries for the last 50 years (5, 6). In contrast, manual laboratory processing of blood cultures is still widespread in many LMICs because it is relatively straightforward and cheap and can be done without the need for expensive and complicated equipment requiring a continuous power supply (3). Like manual culture systems, the blood culture bottles used in automated systems contain a nutrient broth; however, pathogen detection is an automated process, avoiding the need for repeated inspection and subcultures of incubated blood culture bottles by laboratory staff. Sensitivity and time to detection of pathogens of automated systems were previously found to be superior to manual systems (7, 8).

Adequate blood volume is the most important factor determining the detection of microorganisms in blood cultures. This is due to the very low bacterial or fungal density commonly found in the blood of most patients with BSI (9). Overfilling of blood culture bottles can lead to false-positive results irrespective of contamination. This is because the carbon dioxide generated by excessive white blood cells can trigger an alarm in the instrument detectors indicating a positive blood culture (10). A study from Taiwan of 4,844 blood cultures showed that blood volumes of <3, 3–7, 8–10, and >10 mL were associated with bacterial detection rates of 13.31%, 15.02%, 17.68%, and 14.96%, respectively (11). Another very large quality improvement study of 516,201 blood cultures evaluated over 40 months in the USA aimed to improve the percentage of cultures containing the recommended 8–10 mL. After the intervention (education, communication, markings on blood culture bottles), 7/10 participating hospitals collected the recommended ≥8 mL per bottle compared to none before the intervention. The percentage of positive blood cultures increased by 20%, from 7.39% to 8.85% (*P* < 0.001) (12).

Recommendations as to the optimum number of sets of blood cultures vary, but in general, the minimum requirement is one aerobic plus one anaerobic bottle per set in adults (total volume 16–20 mL) and one pediatric bottle for children (total volume 1–3 mL) (13). Anaerobic culture is challenging to set up and frequently not available in LMICs. Currently, most laboratories in Laos are processing a single aerobic bottle per adult patient. The average cost of processing one positive blood culture bottle is around 20 USD, and adding a second bottle would make the test unaffordable (14). This is a concern for individual patient management, since sensitivity of detection will be suboptimal. However, it should have a less important impact on AMR surveillance goals.

In a comparative study from Côte d’Ivoire published in 2021, yield of automated vs manual blood culture was compared by dividing blood from a single venipuncture from 377 patients between manual and automated aerobic blood culture bottles. Automated blood cultures were more likely to be positive than manual blood cultures (36.5% vs 24%, *P* < 0.01 for any growth and 23.3% vs 15.2% for growth with a pathogen). Time to a positive result was shorter using automated processing by approximately 2.5 days (*P* < 0.01) (15). The aim of this study was to compare the yield of aerobic blood culture by manual and automated methods in a hospital in Laos and to explore factors influencing yield, e.g., blood volume (2), age (7), as well as time to report (TTR) a positive result.

## MATERIALS AND METHODS

### Study site and patients

Mahosot Hospital is a primary to tertiary care government teaching hospital with 450 beds, located in Vientiane, Laos. The hospital microbiology laboratory is partly supported by the Lao-Oxford-Mahosot Hospital-Wellcome Trust Research Unit since 2000 and processes around 4,200 blood culture specimens per year from Mahosot Hospital patients. The frequency ranges from 198 to 329 blood cultures per 1,000 admissions annually (Table 1). The laboratory received ISO 15189:2022 accreditation on 25 August 2025 and participates in the United Kingdom External Quality Assessment Service external quality assessment scheme. Blood cultures for Mahosot Hospital patients are provided free of charge, and approximately 70% of patients started on intravenous antibiotics have a blood culture sent to the laboratory (16). The laboratory also receives specimens from other central and provincial hospitals in Laos. Unused blood culture bottles are weighed in the laboratory and the weight written on the label before they are dispatched to the wards and weighed again on arrival in the laboratory. Different digital scales of the same make and model are used for weights pre- and post-sample collection. Scales are calibrated monthly in-house with calibration weights of 100, 10, 1, and 100 mg. Patients who require a blood culture according to their treating clinician are requested to provide written informed consent to agree to their basic demographic data and reason for admission to be recorded and used for research purposes under a protocol investigating the cause of fever in Laos.

**TABLE 1:** Number of blood cultures by year per 1,000 admissions in Mahosot Hospital^*a*^

Year	2018	2019	2020	2021	2022	2023
Number of admissions	19,012	18,392	16,344	11,719	15,256	20,096
Number of blood cultures	3,774	4,955	3,571	2,476	5,017	5,765
Number of blood cultures per 1,000 admissions	198.51	269.41	218.49	211.28	328.85	286.87

^*a*^ The decrease in the number of admissions in 2020–2021 is explained by COVID-19 and the fact that Mahosot Hospital closed beds while moving to a new building on the same site.

Before 8 October 2018, blood culture bottles manufactured by the Laos Pharmacy Factory were processed using conventional manual methods. Two bottles were inoculated per set for children and adults, apart from infants <1 year in whom only one bottle was used. Target volume per bottle varied by age group as follows: 1–2 mL for infants <1 year, 2–4 mL for children >1–15 years, and 5–10 mL for adults >15 years. On 8 October 2018, the laboratory switched to an automated blood culture system (BACTEC FX TOP, BD) and changed to using only one aerobic blood culture bottle in adults. Pediatric bottles are used for children aged less than 15 years. The total target blood volume is 1–3 mL for children 0–15 years and 8–10 mL for adults >15 years. Positive blood cultures detected using the automated system are taken off the machine at various times throughout the day, between 8:00 a.m. and 10:00 p.m. Any positives after 10:00 p.m. are subcultured the following morning at 8:30 a.m. Details for the two blood culture systems are described in Table S1 at https://doi.org/10.6084/m9.figshare.31145131.

Quality control of blood culture media (manual processing) was performed with a positive control and to check for sterility of every batch (see Table S2 at https://doi.org/10.6084/m9.figshare.31145131). Nine blood culture bottles were selected at random from different age groups, such as infants, children, and adults. Secondly, three unused blood culture bottles were processed as a sterility test (negative control). Thirdly, as a positive control, two-blood culture bottles were inoculated with 500 µL of 0.5 McFarland standard of each of *Streptococcus pneumoniae* (NCTC 12977), *Pseudomonas aeruginosa* (ATCC 2753), and *Haemophilus influenzae* (NTCT 11931) in accordance with the SOP at that time. All blood culture bottles were incubated at 37°C for 7 days, read by visual inspection every day. If a blood culture became cloudy, the subculture would be inoculated on blood, MacConkey, and chocolate agar. On every VITEK MS run, we incorporated one ATCC 8739 strain of *Escherichia coli* as QC.

Data on patients who had blood cultures taken from 1 January 2016 to 31 December 2023 were extracted into a Microsoft Excel database from the Laboratory Information Management System and a research study database.

The blood volume was estimated from the bottle weight after blood inoculation, minus the bottle weight pre-sampling and plus 0.41 g to account for the weight of the bottle cap for automated cultures and 0.04 g for manual cultures. Bottle cap weight was estimated by taking the average weight of 48 caps from BACTEC bottles and 30 caps from manual bottles.

We calculated the time to report (TTR) for the first positive blood culture in days by subtracting the date the sample was received in the laboratory from the date of the first subculture with a Gram stain showing the organism. This was chosen in preference to time to flag positive as a more clinically relevant comparable measure between the two culture methods. It was not possible to distinguish recovery of pathogens by blind subculture vs due to cloudy broth since these data were not captured in the database.

Positive blood cultures were processed following local standard operating procedures. In brief, manual blood cultures were checked daily for visible growth (cloudy broth), and a Gram stain and subculture were performed if considered positive. Blind subculture was performed on days 1, 3, 5, and 7. For the automated system, bottles were incubated for 5 days. If a bottle flagged positive, they had a Gram stain performed and were subcultured onto the appropriate agar. If this occurred at night, the Gram stain was delayed until the following morning. All subcultures were incubated at 37°C in air with plates checked daily for growth. Identification methods included biochemical testing, API (bioMérieux), or matrix-assisted laser desorption ionization-time of flight mass spectrometry (VITEK MS, bioMérieux). All isolates were submitted to API or to the VITEK MS after 2021. *Salmonella* Typhi was identified using serological/agglutination methods and API 20E.

A blood culture episode was defined as all blood culture specimens taken within two calendar weeks from a single patient growing the same organism. Results are reported following the MICRO Checklist (see Table S3 at https://doi.org/10.6084/m9.figshare.31145131) (17).

### Definitions

Organisms regarded as contaminants for this analysis included coagulase-negative staphylococci, *Streptococcus mitis* group (with exceptions, e.g., patients with suspected bacterial endocarditis), *Corynebacterium* spp. or diphtheroids (not *Corynebacterium diphtheriae*), *Bacillus* spp. (not *Bacillus anthracis*), *Micrococcus* spp., *Burkholderia cepacia* complex, and *Pseudomonas* spp. (non-aeruginosa) (18–24).

Underfilled blood culture volume was defined as the blood sample below the minimum recommended volume. Overfilled blood culture volume meant the blood sample exceeded the maximum recommended volume (25).

### Statistical analysis

Continuous data with skewed distribution, such as age and volume of blood, were summarized using medians and interquartile range (IQR). If blood volume was less than zero (attributed to error and/or variation in scales used for pre- and post-weights), then median volume for each age group was imputed. We performed a sensitivity analysis, in which missing data were excluded (i.e., complete case analysis). Proportional data were summarized using frequencies and percentages. A multivariable logistic regression model was fitted to assess the factors that were independently associated with the growth of contaminant or significant bacterial isolates. The odds ratios (ORs) and the corresponding 95% confidence intervals (CIs) were reported for these models. The time to report a culture with a significant pathogen was assessed using the survival analysis method. In this analysis, a Kaplan-Meier plot was done to show the differences in the time to report significant growth over the observation period. Statistical comparisons of the time to report a culture were performed using the Cox proportional hazards model. The hazard ratios (HRs) and the corresponding 95% CIs have been reported for this model. The main comparisons of interest were between the manual and the automated systems.

Fisher’s exact test was used to compare detection of clinically significant bacterial isolates vs presumed contaminants in adults and children using both blood culture methods. A *P* value below 0.05 was considered statistically significant. Data analysis was performed using Stata version 14.1.

## RESULTS

**Fig 1:**
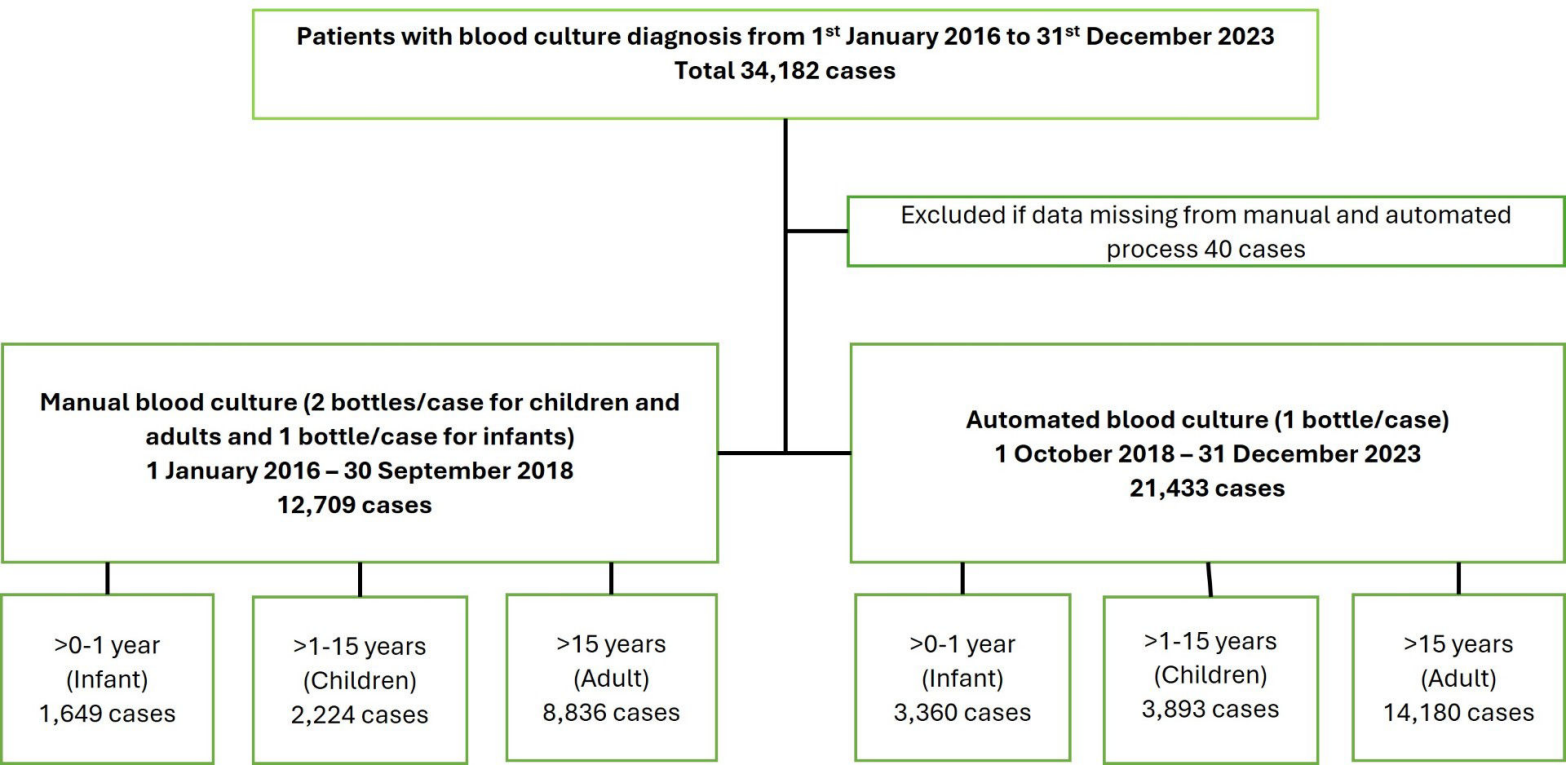
Study flow diagram.

A total of 34,182 patients had a blood culture between 1 January 2026 and 31 December 2023; 40 cases were excluded because of missing data (Fig. 1). There were 12,709 cases who had blood cultures processed manually from 1 January 2016 to 7 October 2018 and 21,433 cases from 8 October 2018 to 31 December 2023 who had blood culture processed in an automated system. All blood cultures from Mahosot Hospital received in the laboratory were included in the study, including sequential blood cultures from the same patient. The average number of admissions from 1 January 2018 until 31 December 2023 was 16,803 cases per year. The median (range) number of blood cultures sent to the microbiology laboratory per year between 2018 and 2023 was 4,260 (2,476–5,765) (Table 1).

Age and gender distributions were generally similar between manual and automated groups (Table 2). The median blood volume per blood culture set was 8.6 mL (IQR 2.0–11.4) for those processed by automated methods (baby, 0.9 mL; children, 1.8 mL; and adult, 10.6 mL) and 10.5 mL (IQR 4.9–11.5) for manual methods (baby, 1.9 mL; children, 4.5 mL; adult, 11.2 mL). Blood volume data by age group for manual and automated methods are presented in Table S4 at https://doi.org/10.6084/m9.figshare.31145131. Data were excluded if blood volume was missing (176 cases). For manual blood culture exclusions, this included 14 infants, 23 children, and 100 adults. For automated blood culture exclusions, this included five infants, 10 children, and 24 adults. The analysis was repeated using the imputed median blood volume for each age group and was similar to those obtained without imputation (see Table S5 at https://doi.org/10.6084/m9.figshare.31145131). Blood culture volumes were missing for 137 cases of manual blood culture and 39 cases of automated blood culture. The percentage of significant pathogens and presumed contaminants did not differ between the manual and automated systems (Table 2). The results of the analysis using the specimens excluded due to missing blood volume, along with the effects of age and blood volume on the detection of significant and contaminant organisms, are presented in Table 3. For every 1 mL increase in blood volume, growth with a significant pathogen increased by 2.3%, while growth with a presumed contaminant decreased by 1.3%. Additionally, for each 1-year increase in age, growth with a significant pathogen increased by 1.6%. The automated blood culture process was associated with a 2.3% reduction in growth with presumed contaminants compared to the manual process.

**TABLE 2:** Characteristics of patients who had blood cultures during the study period

Characteristics	Manual Two bottles/set for >1–15 years and one bottle/set for >0–1 year				Automated One bottle/set				*P* value
	>0–1 year	>1–15 years	>15 years	Total	>0–1 year	>1–15 years	>15 years	Total	
Age (years), median (IQR), *N* (%)	6 days (1 day–3 months), 1,649 (13.0)	6.0 (3.0–11), 2,224 (17.5)	46.0 (30–62), 8,836 (69.5)	31 (10–56 years), 12,709 (100)	5 days (1 day–4 months), 3,360 (15.7)	6.0 (2.9–10), 3,893 (18.2)	48.0 (30–62), 14,180 (66.1)	30 (7–55 years), 21,433 (100)	<0.001
Gender, *N* (%)									<0.001
Male	910 (55.2)	1,277 (57.4)	4,839 (54.8)	7,026 (55.3)	1,894 (56.4)	2,156 (55.4)	7,333 (51.7)	11,383 (53.1)	
Female	739 (44.8)	947 (42.6)	3,997 (45.2)	5,683 (44.7)	1,466 (43.6)	1,737 (44.7)	6,847 (48.3)	10,050 (46.9)	
*N*	1,649	2,224	8,836	12,709	3,360	3,893	14,180	21,433	
Volume of blood per set (mL), median (IQR), *N*	1.9 (1.5–2.4), 1,635	4.5 (3.7–5.2), 2,201	11.2 (10.3–11.8), 8,736	10.5 (4.9–11.5), 12,572	0.9 (0.7–1.4), 3,355	1.8 (1.2–2.7), 3,883	10.6 (8.5–13.1), 14,156	8.6 (2.0–11.4), 21,394	<0.001
Growth (per case)									0.156
No growth, *N* (%)	1,454 (88.2)	2,052 (92.3)	7,827 (88.6)	11,333 (89.2)	2,927 (87.1)	3,571 (91.7)	12,756 (90.0)	19,254 (89.8)	
Growth with significant pathogen, *N* (%)	54 (3.3)	62 (2.8)	739 (8.4)	855 (6.7)	113 (3.4)	105 (2.7)	1,121 (7.9)	1,339 (6.3)	
Growth with presumed contaminant, *N* (%)	141 (8.5)	110 (4.9)	270 (3.0)	521 (4.1)	320 (9.5)	217 (5.6)	303 (2.1)	840 (3.9)	

**TABLE 3:** The effects of blood volume and blood culture process on detection of significant and presumed contaminant organisms (blood volume missing data excluded)

	Multivariable odds ratio	*P* value	95% CI
Growth with significant pathogen			
Blood volume (mL)	1.023	<0.001	1.013–1.033
Automated	0.966	0.448	0.882–1.057
Age (year)	1.016	<0.001	1.014–1.018
Growth with presumed contaminant			
Blood volume (mL)	0.870	<0.001	0.854–0.886
Automated	0.769	<0.001	0.684–0.864
Age (year)	1.003	0.031	1.000–1.006

The top three significant pathogens in children (≤15 years) were *Staphylococcus aureus*, *Escherichia coli*, and *Burkholderia pseudomallei*, and those in adults were *E. coli*, *B. pseudomallei*, and *Klebsiella pneumoniae* (see Table 4 for the top five significant pathogens in all age groups combined). The most common significant pathogen in neonates and infants was *E. coli*, accounting for 13 cases (24.1%) from the manual method and 8 cases (7.1%) from the automated method (Table S6 at https://doi.org/10.6084/m9.figshare.31145131). The top three presumed contaminants were coagulase-negative staphylococci, *Bacillus* species, and *Corynebacterium* species in both manual and automated blood cultures. A complete list of all organisms isolated can be found in Tables S6 to S8 at https://doi.org/10.6084/m9.figshare.31145131. Using manual methods, a total of 186 organisms were cultured after day 5. Of these 73 bacteria, 26 *Talaromyces* spp. and 10 *Cryptococcu*s spp. were classified as significant pathogens, and 77 bacteria were classified as contaminants (Table S9 at https://doi.org/10.6084/m9.figshare.31145131). Matrix-assisted laser desorption ionization-time of flight was used to identify 7% of organism isolates, with the remainder identified using biochemical methods.

**TABLE 4:** Five most common significant pathogens and presumed contaminants isolated in manual and automated blood cultures

Bacterial organism	Manual, *N* (%) 1 January 2016–7 October 2018	Automated, *N* (%) 8 October 2018–31 December 2023	Total *N* (%)	*P* value
Significant growth	Total positive = 855	Total positive = 1,339	Total positive = 2,194	
*Escherichia coli*	235 (27.49)	306 (22.85)	541 (24.66)	0.028
*Burkholderia pseudomallei*	140 (16.37)	253 (18.89)	393 (17.91)	0.112
*Klebsiella pneumoniae*	66 (7.72)	126 (9.41)	192 (8.75)	0.316
*Staphylococcus aureus*	51 (5.96)	114 (8.51)	165 (7.52)	0.031
*Salmonella* Typhi	52 (6.08)	19 (1.42)	71 (3.24)	<0.001
Presumed contaminant	Total positive = 521	Total positive = 840	Total positive = 1,361	
Coagulase-negative staphylococci	138 (26.4)	464 (55.24)	602 (44.23)	<0.001
*Bacillus* species	74 (14.20)	76 (9.45)	150 (11.02)	0.009
*Corynebacterium s*pp.	19 (3.65)	49 (5.83)	68 (5.00)	0.045
*Streptococcus mitis* group	18 (3.45)	30 (3.57)	48 (3.53)	0.881
*Burkholderia cepacia* complex	4 (0.77)	7 (0.83)	11 (0.81)	0.687

Blood volume, age, and the type of blood culture and processing method (manual or automated) were independently associated with detecting growth of a significant pathogen (Table 3). The odds ratio of detecting growth of a significant pathogen increased by about 2.3% per 1 mL increase in blood volume (OR = 1.023, 95% CI [1.013–1.033], *P* < 0.001). Underfilling of automated system blood culture bottles was associated with decreased growth of significant pathogens in all age groups (see Table S10 at https://doi.org/10.6084/m9.figshare.31145131).

Similarly, blood volume and the type of blood culture (manual or automated) were independently associated with detecting growth of contaminants (Table 3). However, there was an inverse relationship for the blood culture method and blood volume. Conversely, the odds of detecting growth of a contaminant decreased by about 13% per 1 mL increase in blood volume (OR = 0.870, 95% CI [0.854–0.886], *P* < 0.001). The odds of detecting growth of a contaminant decreased by about 23% with the automated blood culture process compared to the manual one (OR = 0.769, 95% CI [0.684–0.864], *P* < 0.001). Results were similar if we imputed the median blood volume of each age group for missing data using the manual as the reference (see Table S5 at https://doi.org/10.6084/m9.figshare.31145131).

**Fig 2:**
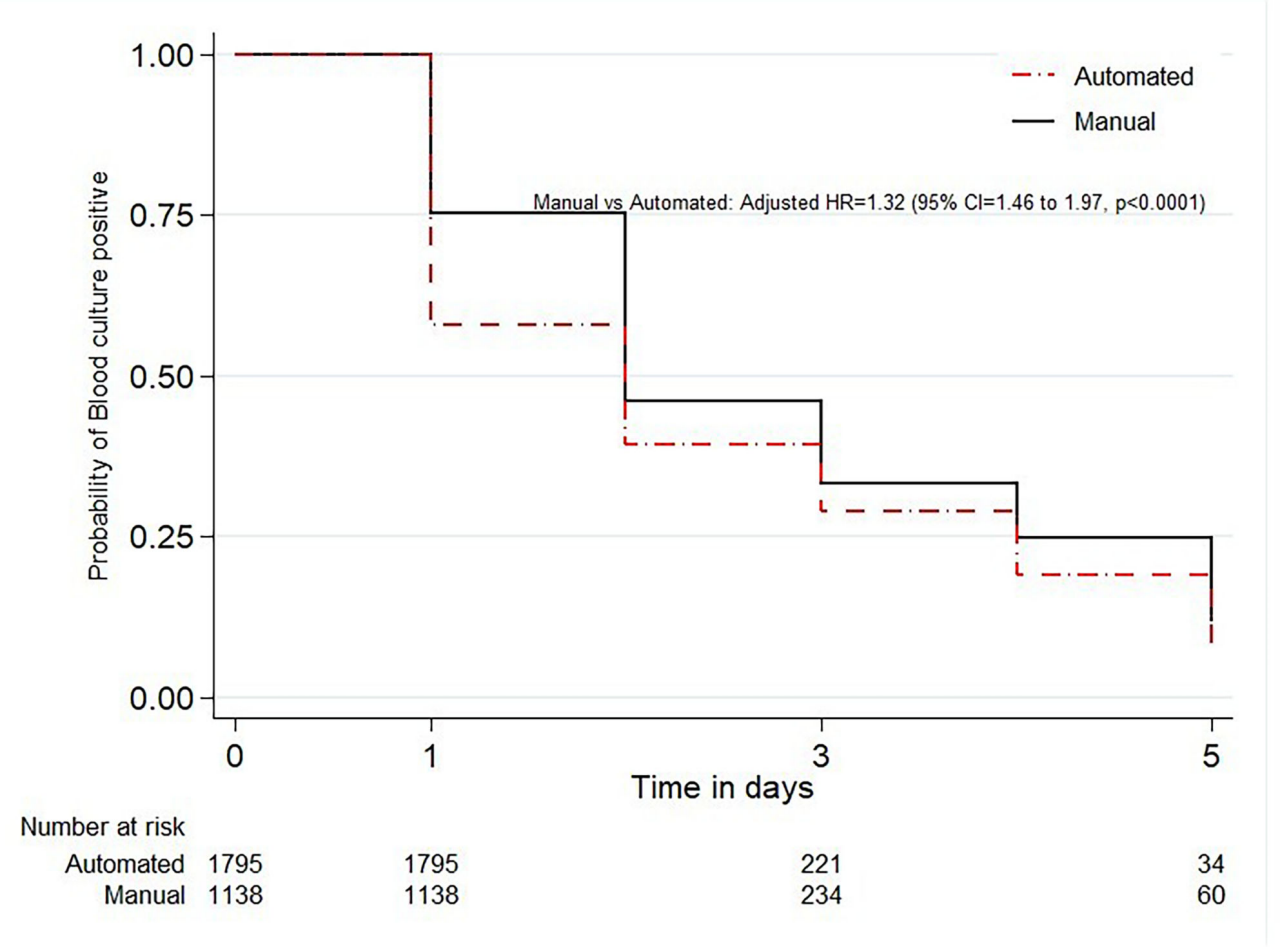
Time to isolation of a significant pathogen using manual and automated blood culture processing methods.

The day of detection was taken into account in the Kaplan-Meier survival analyses (Fig. 2; see also Fig. S1 at https://doi.org/10.6084/m9.figshare.31145131). The time taken to report growth with a significant pathogen for the automated process was approximately 1.3 times faster than the manual one (HR = 1.32, 95% CI [1.20–1.46]) (Fig. 2). The median time to report significant growth was 2 days for the automated process (IQR = 1–4 days) and 2 days for the manual process (IQR = 2–4 days) within 5 days. The manual method was associated with detection of a slightly higher percentage of contaminants (*P* < 0.001). The median (range) time to growth of a presumed contaminant using manual methods was 5 (2–9) days and that using automated methods was 2 (1–5) days (see Fig. S2 at https://doi.org/10.6084/m9.figshare.31145131). As shown in Fig. 2, the automated method led to faster reporting of detection of significant pathogens than the manual method.

## DISCUSSION

The need for more globally representative AMR surveillance data has led to strengthening of diagnostic microbiology in several LMICs. While high-income countries have embraced automation in diagnostic microbiology laboratories for decades, this has been adopted unevenly in LMICs due to barriers such as cost, unreliable consumable supply chain, and unstable electricity supply. In many LMICs, diagnostic microbiology tests are performed in the general hospital laboratory, which is frequently short staffed, with staff rotating between blood sciences, blood bank, and microbiology duties. The COVID-19 pandemic saw laboratory technicians diverted away from routine activity, especially microbiology, to implement SARS-CoV-2 molecular diagnostics. With automated blood culture systems, the need for daily inspection and subculture is removed, enabling staff to get on with other tasks. Since 2022, the Fleming Fund has supported the introduction of an automated blood culture system (BACTEC FX 40) in several provinces in Laos, in an effort to strengthen global AMR surveillance. Other approaches targeting LMIC settings are under evaluation, e.g., the MiniLab created by Médecins Sans Frontières, which uses manual blood culture methods and biphasic media to facilitate subculture (26).

Several studies have shown that bacterial growth from a blood culture can be influenced by many factors, including bacterial load in the patient, blood volume (27), and blood culture method (28). In Laos, a single-sampling strategy using one aerobic blood culture bottle for most culture requests has been adopted, which is easier and cheaper than the multisite sampling and larger volume blood draw that are typically recommended, although a suboptimal sensitivity is anticipated compared to the accepted gold standard of sending one to two sets per patient (29). Switching from a manual to an automated blood culture system did not lead to an increased yield of positive cultures as we anticipated and as shown in other studies. We detected a difference in age and gender between manual and automated groups that reached statistical significance due to the large sample size; however, the small differences are not of clinical importance. The most likely explanation for the lack of increase in culture yield is the drop in average blood volume per specimen in all age groups after introducing the automated system. The average blood volume taken for blood culture was 11.7 and 10.5 mL for adults, 5.0 and 1.3 mL for children; and 2.3 and 0.9 mL for infants for manual and automated systems, respectively. The drop in blood volume sent, particularly in the 1- to 15-year age group, when we switched to an automated system, likely reflects the change to using a single bottle per set from two bottles for adults and children, although the volume taken from infants also dropped. The BACTEC pediatric bottle has 1–3 mL written on the side, which may have led staff to consider that 1 mL was acceptable. Another explanation could be higher rates of antibiotic pre-treatment. In a study in Germany, blood culture positivity was two times higher among patients with suspected sepsis who did not receive antibiotics than those who were already receiving antibiotics (*P* value < 0.001) (27, 30). It has been shown that antibiotic administration in the week prior to bacteremia can delay or reduce microorganism growth (*P* < 0.001) (27, 31). In Lao, self-medication with antibiotics is common, as shown in previous studies (32), and children tend to receive antibiotics more frequently than adults (63% vs 47%, *P* < 0.00010) (33).

Our study found that bacterial growth was significantly lower in children than in adults, while presumed contaminants were more frequently identified in children than in adults, a trend consistent with other studies (34, 35). The blood volume used was based on the manufacturer’s recommendations and pediatric wards in the hospital only stock pediatric blood culture bottles. Median weight of hospitalized Lao children aged 12–15 years from previous studies was 42.20 kg. According to the Seattle Children’s Hospital guidelines for maximum allowable blood draw volumes in children, children weighing 41–45 kg can have up to 82–90 mL blood drawn (36). While there is no consensus on the optimum blood volume for culture in children (37), using adult blood culture bottles for older children could be considered, since collecting multiple blood cultures with age-appropriate volumes before starting antibiotics increases pathogen detection rates in children (38). The TTR positive blood cultures was longer in children aged 12 months and older, potentially due to the blood culture sample volume (39). Smaller blood volumes can reduce the sensitivity of blood cultures, resulting in fewer pathogens and a higher risk of contamination (40, 41). In our study, the number of pathogens isolated was similar between manual and automated blood culture methods for children, as the blood volume in the BACTEC system was lower than the expected range. However, several pediatric studies have indicated that BACTEC yields higher blood culture results, with some reporting a yield up to five times greater than conventional methods (28, 42).

Our study found that *Bacillus* species was detected 1.50 times more frequently in a set of two blood culture bottles processed manually than one automated set. The fact that the median time to isolate a presumed contaminant organism was 3 days longer with the manual method could indicate contamination due to repeated subculture. A closed system for blood drawing provides more safety and decreases contamination from the environment compared to open systems, where repeated subcultures are performed (43). We also switched to a vacutainer for venipuncture when the automated system was introduced. Several studies have shown that a 5-day incubation time can reduce the recovery of contaminants (44) and improve detection rates (44, 45).

We found automated blood culture processing reduced time to report growth with a significant pathogen by 1.3 times compared to manual processing (*P* < 0.001). This finding was consistent with the results reported by Surase et al. in 2016 (46). Improving turnaround times is an important goal to support antimicrobial stewardship. While we did not find an improved pathogen yield after switching to the automated system—which may be related to the blood volume inoculated, prior treatment with antibiotics, or other factors—overall pathogen recovery was similar, and the automated method is lower cost if only one bottle is used and requires less staff time for processing (see Table S11 at https://doi.org/10.6084/m9.figshare.31145131).

Adding a second culture bottle is appealing, but it would double the cost. In an economic evaluation of diagnostic microbiology, the expenditure per specimen using BACTEC without automated AST was determined to be 21.95 USD (based on an annual processing of 10,000 specimens). Material costs constitute the majority of the expense (between 69% and 90%), followed by labor costs (between 10% and 31%), with no capital costs at either workload level. The corresponding cost per isolate ranged from 215 USD to 304 USD (14). This cost range is prohibitive for many low-resource settings, where, in the absence of donor funding, patients often bear the burden of laboratory test expenses. On a positive note, while the automated blood culture process used less blood overall, yield of significant bacteria was similar to manual methods with a larger volume of blood processed.

Limitations of this study include the fact it was not randomized, and the manual and automated methods were implemented sequentially rather than in parallel. We also did not have data on prior antibiotic use, which would also affect yield of a positive result. It is also possible that the COVID-19 pandemic impacted our findings (47). While time to report was a clinically relevant measure in our setting, ideally time to flag positive would be compared since this is likely to be an advantage of automated systems in laboratories with 24 h processing of blood cultures that may lead to a clinically important shortening of the time to deliver results to clinicians managing patients. During the period using manual methods, the subculture was done after 24 h of bottle incubation, but this was recorded in the logbook, not in the database.

### Conclusion

In a primary to tertiary hospital in Laos, automated processing of blood cultures did not impact the yield of significant growth but did reduce the time to report growth with a significant pathogen compared to manual processing. However, switching to a single bottle led to a reduction in the total volume of blood being sent to the laboratory for culture in all age groups. Monitoring and feedback to clinicians on blood volume received may help optimize the use of blood culture. Adding another aerobic or anaerobic culture bottle for adults would be desirable but would double the cost. Subsidized prices for bacteriology consumables for low-resource settings would increase equitable access to diagnostic microbiology.

## Data Availability

The Supplemental material file is available in the figshare repository at https://doi.org/10.6084/m9.figshare.31145131. Data are available on request from the MORU Data Access Committee (datasharing@tropmedres.ac).
